# Effects of a One Year Reusable Contraceptive Vaginal Ring on Vaginal Microflora and the Risk of Vaginal Infection: An Open-Label Prospective Evaluation

**DOI:** 10.1371/journal.pone.0134460

**Published:** 2015-08-12

**Authors:** Yongmei Huang, Ruth B. Merkatz, Sharon L. Hillier, Kevin Roberts, Diana L. Blithe, Régine Sitruk-Ware, Mitchell D. Creinin

**Affiliations:** 1 Population Council, Center for Biomedical Research, New York, United States of America; 2 Department of Obstetrics, Gynecology and Reproductive Sciences, University of Pittsburgh and the Magee-Womens Research Institute, Pittsburgh, United States of America; 3 Contraceptive Discovery and Development Branch, National Institute of Child Health and Human Development, Bethesda, United States of America; 4 Department of Obstetrics and Gynecology, University of California Davis, Sacramento, United States of America; Rush University, UNITED STATES

## Abstract

**Background:**

A contraceptive vaginal ring (CVR) containing Nestorone® (NES) and ethinyl estradiol (EE) that is reusable for 1- year (13 cycles) is under development. This study assessed effects of this investigational CVR on the incidence of vaginal infections and change in vaginal microflora.

**Methods:**

There were 120 women enrolled into a NES/EE CVR Phase III trial and a microbiology sub-study for up to 1- year of cyclic product use. Gynecological examinations were conducted at baseline, the first week of cycle 6 and last week of cycle 13 (or during early discontinuation visits). Vaginal swabs were obtained for wet mount microscopy, Gram stain and culture. The CVR was removed from the vagina at the last study visit and cultured. Semi-quantitative cultures for *Lactobacillus*, *Gardnerella vaginalis*, *Enterococcus faecalis*, *Staphylococcus aureus*, *Escherichia coli*, *anaerobic gram negative rods* (GNRs), *Candida albicans* and other yeasts were performed on vaginal and CVR samples. Vaginal infections were documented throughout the study.

**Results:**

Over 1- year of use, 3.3% of subjects were clinically diagnosed with bacterial vaginosis, 15.0% with vulvovaginal candidiasis, and 0.8% with trichomoniasis. The detection rate of these three infections did not change significantly from baseline to either Cycle 6 or 13. Nugent scores remained stable. H_2_O_2_-positive *Lactobacillus* dominated vaginal flora with a non-significant prevalence increase from 76.7% at baseline to 82.7% at cycle 6 and 90.2% at cycle 13, and a median concentration of 10^7^ colony forming units (cfu) per gram. Although anaerobic GNRs prevalence increased significantly, the median concentration decreased slightly (10^4^ to 10^3^cfu per gram). There were no significant changes in frequency or concentrations of other pathogens. High levels of agreement between vaginal and ring surface microbiota were observed.

**Conclusion:**

Sustained use of the NES/EE CVR did not increase the risk of vaginal infection and was not disruptive to the vaginal ecosystem.

**Trial Registration:**

ClinicalTrials.gov NCT00263341, NCT00455156

## Introduction

The Population Council is an international non- governmental research organization that was started in the mid -1950s to conduct research and address critical health and development issues in low resource settings. In the field of reproductive health, the Council has focused on development of long-acting, reversible contraceptives including the Copper T intrauterine device (IUD), the levonorgestrel intrauterine system (Mirena®), and implants such as Jadelle® and Norplant®. Despite a worldwide increase in contraceptive use, the unmet need for additional modern methods has intensified due to population growth, ongoing access issues, and an increase in global awareness and commitments [1,2]. In response, and with support and collaboration from the United States Agency for International Development, the National Institutes of Health (NIH), the World Health Organization, and the Bill and Melinda Gates Foundation, the Council has been developing a contraceptive vaginal ring (CVR) made of silicone that is designed to provide contraceptive protection during one year of use [3–5]. The CVR contains Nestorone® (NES), a new 19-nor progesterone derivative without androgenic activity, and a low dose of ethinyl estradiol (EE) [3–5]. The goal is to offer a safe, effective, acceptable long-acting user controlled reversible contraceptive that can be inserted and removed by the woman herself rather than by specially trained health care providers, does not require daily action, and can be reused up to one year (13 cycles). It is designed so that refrigeration is not required. Such features may improve overall method adherence and address access and service delivery issues [3–5], especially in low resource settings where health resources are limited and the unmet need for contraception remains high [1,2] In addition to assessing the overall safety, efficacy, and acceptability of this novel CVR, effects of cyclic use of a single vaginal ring used repeatedly for a full year warranted evaluation, specifically its impact on the incidence of vaginal infections and the vaginal microbiota.

The normal vaginal ecosystem can be disrupted by a variety of factors including hormonal fluctuations, sexual activity (frequency and number of partners), and use of vaginal products, antibiotics, and douching [6–8]. Reports from various CVR studies that have examined symptoms associated with ring use including vaginal wetness and leukorrhea [9–11], have not demonstrated an overgrowth of pathogenic organisms or an increased incidence of symptomatic vaginal infections [12–14]. Trials of CVRs containing levonorgestrel and estradiol (one ring used cyclically for six months) or etonogestrel and EE (one ring used for either 21, 28, 42, or 56 days) demonstrated a good safety profile relative to vaginal microflora [12–14]. Two additional studies that compared the CVR containing etonogestrel and EE (NuvaRing®, Merck & Co. Inc, Roseland, NJ) with an oral contraceptive regimen demonstrated that use of this monthly ring resulted in higher mean concentrations of *Lactobacillus* as detected by culture and Gram stain [15–16]. All of these CVR studies, however, have focused on relatively short-term usage of a single ring while the investigational NES/EE CVR is intended for a full year’s use. Earlier studies with this CVR did not suggest any safety concerns related to vaginal infections; however a focused microbiology study had not been conducted. Therefore, we conducted a safety substudy nested in a large Phase III study to assess the occurrence of common vaginal infections, specifically bacterial vaginosis (BV), trichomoniasis, and vulvovaginal candidiasis (VVC) during cyclic use of a single NES/EE CVR for up to one year. Secondarily, we evaluated changes in selected components of the vaginal microbiota during ring use. We assessed the frequency and concentration of key microbes associated with indicators of vaginal health (*Lactobacillus* species), BV (*Gardnerella vaginalis* and anaerobic gram negative rods), urinary tract infections/UTIs (*Escherichia coli*), VVC (*Candida*. *albicans* and other yeast species), and toxic shock syndrome (*Staphylococcus*. *aureus*).

## Methods

### Clinical Study

The Phase III trial of the NES/EE CVR was originally posted to clintrials.gov on December 6, 2005 (Number: NCT00263341). The National Institute of Child Health and Human Development (NICHD/NIH was listed on this posting as a collaborator in conducting the study. Additional FDA requirements received after this date required changes to the protocol and the addition of more sites, which was then posted again to clintrials.gov on April 2, 2007 (Number: NCT00455156). All subjects were enrolled after the original posting on December 6, 2005.

**Fig 1:**
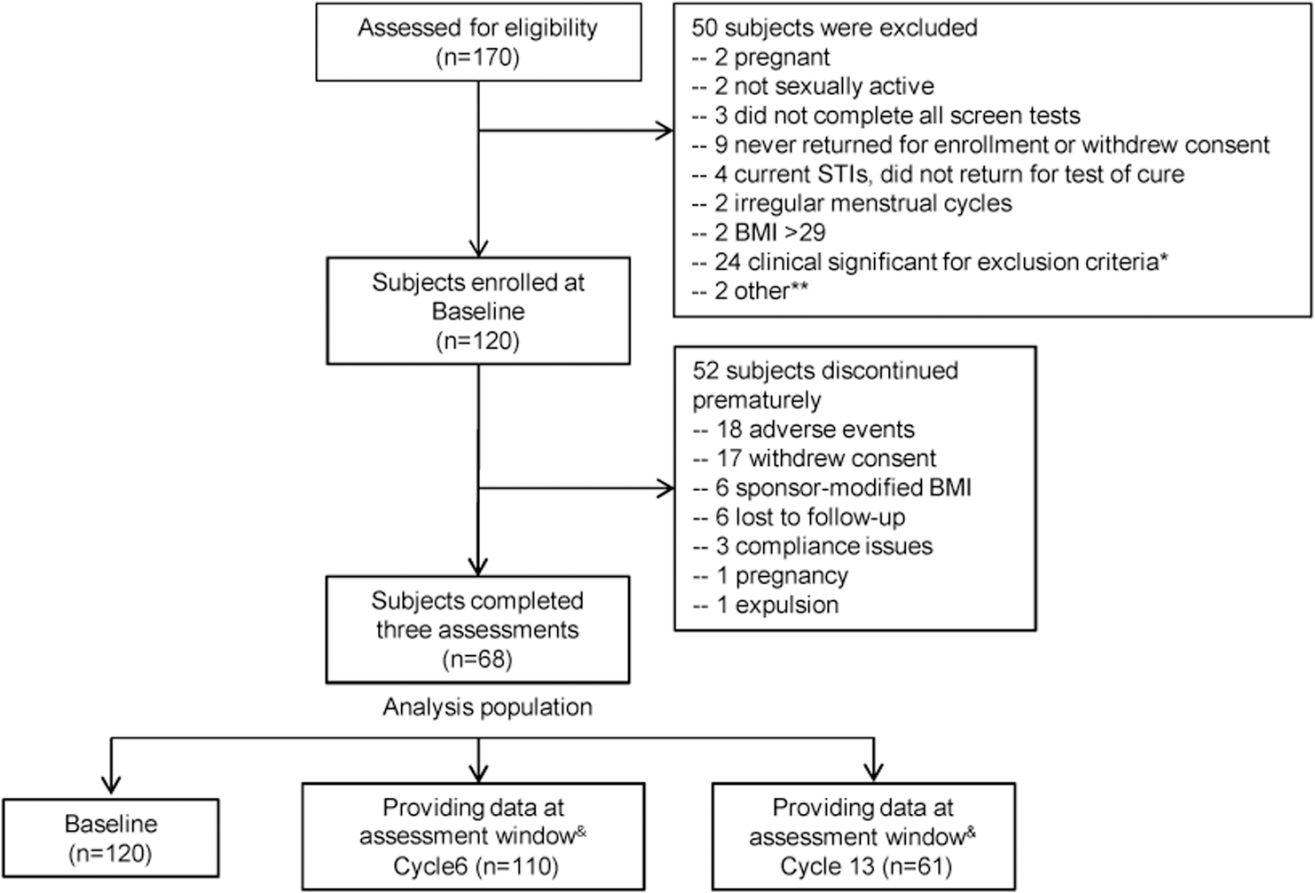
Subjects’ disposition chart in microbiology study. * Thirteen cases of abnormal pap smear, one current PID, one vaginal cyst, three migraines or headaches, one heavy smoking, one drug abuse, two hypertension, one abnormal blood cholesterol, one abnormal urine test. ** One in another study, one employed in the office. ^&^Visit timing: Cycle 6 (targeted at Day 144 [beginning of Cycle 6], range 2 to 251); Cycle 13 (targeted at Day 358 [12 cycles plus 21 days of use during cycle 13], range 252 to 358).

The study protocol, the TREND checklist, and the flow chart are available as supporting information; see S1 Protocol, S1 Checklist, and Fig 1. There were no changes to the microbiology substudy protocol during the course of the investigation. The authors confirm that all ongoing and related trials for NES/EE CVR are registered on clinicaltrials.gov.

### Ethics

This study was conducted at Magee-Womens Hospital of the University of Pittsburgh Medical Center between January 2007 and January 2009. Women in this study were recruited from the University setting and from Pittsburgh metropolitan area for participation in the pivotal Phase III multicenter, open-label trial of the efficacy, cycle control and safety of the NES/EE 150mcg/15mcg CVR that was conducted at fifteen U.S. sites and supported by the NICHD at the NIH and the Population Council. Subjects at the Pittsburgh site were recruited for participation in both the Phase III study and the microbiologic substudy. Both studies were approved by Chesapeake Research Review Inc, the Institutional Review Boards of the NICHD Coordinating Center (October 13, 2006), the Population Council (October 18, 2006), and the University of Pittsburgh (November 14, 2006). This study was conducted in accordance with the Declaration of Helsinki and Good Clinical Practice guidelines.

### Study Conduct and Population

Recruitment into the Phase III study and the microbiology safety substudy was conducted from February 2007 through May 2008. Healthy, sexually active women at least 18 and less than 40 years of age with a history of regular menstrual cycles of 21 to 35 days' duration were screened for admission into the Phase III study and this microbiology safety substudy. All participants signed an informed consent for the safety and efficacy study and the substudy. In accordance with the Phase III protocol requirement, participants were excluded if they had contraindications to hormonal contraceptive use or silicone rubber, or if their weight was > 90 kg. Due to safety concerns related to EE and the risk for venous thrombotic events in heavy/obese women, six months after the trial began the Data Safety Monitoring Board that was established for the large Phase III clinical trials recommended that we exclude women with a body mass index (BMI) > 29.0. Women who had been enrolled with BMIs > 29.0 were, therefore, discontinued. Other entry exclusions included clinically significant abnormalities related to vital signs, blood chemistries and hematologic parameters, and results of physical and gynecologic exams including Pap smear testing suggestive of significant health issues. Participants with known HIV infection or at high risk of acquiring HIV were not included in the study; however, those with vaginal infections or sexually transmitted infections (STIs) diagnosed at screening including chlamydial cervicitis and gonococcal cervicitis were eligible following effective treatment.

Eligible subjects were scheduled to return to the investigational site six times over one year of CVR use. The initial visit occurred during days 2–5 of each participant’s first menses following screening at which time enrollment criteria were confirmed. The NES/EE CVR was then dispensed by a clinician designated in accordance with regulatory requirements as a clinical provider for this study, and participants inserted the ring. Subjects were counseled to use the ring for 21 consecutive days and remove it for 7 days, followed by reinsertion of the same ring to begin the next cycle. The same ring was used repeatedly for a full year. The women were instructed that upon removal at each cycle, and as part of routine hygiene they were to wash the ring with mild soap and lukewarm water, dry the ring and store it in a small plastic case at room temperature and away from direct sunlight. Participants were given daily diaries to record dates and times when the ring was out of the vagina, dates when they experienced any bleeding or spotting, ring expulsions, had sexual intercourse, encountered problems with ring use or had medical issues and used medications.

After enrollment, participants were scheduled to return during the first week of cycles 3, 6, and 9, and on day 21–23 of cycle 13 (exit visit) for pregnancy testing, vital signs and weight measurements. They were queried about the occurrence of any problems associated with ring use or adverse events (AEs), including symptoms of vaginal infections, which may have prompted further clinical assessments or medical treatments including use of concomitant medications. Laboratory safety assessments completed at screening were repeated at cycle 6 and at the exit visit. Participants returned for a post-treatment visit within 1–2 weeks from their exit visit for pregnancy testing, vital signs and weight measurements, and to check on the occurrence of AEs. Throughout the study, any symptomatic diagnosis of BV, VVC, or other vaginal infections was treated with oral antibiotics or antifungals. Participants were followed through December 31, 2008 (or early January 2009 in a few instances). They were encouraged to maintain the visit schedule; visit window deviations or other deviations were recorded. They were given a small stipend to cover travel and any child care costs incurred during study visits.

### Microbiology Study Assessments

In addition to the routine Phase III study evaluations, substudy participants had other assessments as part of the vaginal examination at screening, and at the cycle 6 and 13/exit visits. In accordance with standard clinical and laboratory procedures, wet mounts were prepared for evaluation of pH, BV (using Amsel criteria), and trichomoniasis. A potassium hydroxide (KOH) slide preparation was utilized for diagnosing VVC. Gram stained vaginal smears were prepared for laboratory evaluation/assignment of the Nugent score and a semi-quantitative assessment for the absence or presence of neutrophils. A Nugent score of 0–3 was interpreted as normal, *Lactobacillus*-predominant flora, 4–6 corresponded to intermediate flora, and 7–10 was indicative of BV [17]. In addition, two sterile vaginal swabs were collected, placed in anaerobic transport media (Port-a-cul tube) and transported to the laboratory within 24 hours for culture detection of hydrogen peroxide (H_2_O_2_) positive *Lactobacillus*, H_2_O_2_-negative *Lactobacillus*, *Gardnerella vaginalis*, *Enterococcus faecalis*, *Staphylococcus aureus*, *Escherichia coli*, *anaerobic gram negative rods (GNRs)*, *Candida albicans*, and other yeast. Although anaerobic gram negative rods include microbes from different genera that are not all associated with BV to the same degree, individual species were grouped together for the purpose of this study [18]. At the cycle 13 or early discontinuation visit, the study ring was removed by the study clinician using sterile gloves, put into a transport tube and sent to the lab overnight. The ring surface was cultured and the swab processed in parallel with the vaginal swab sample for the same organisms.

The swabs were used to inoculate Columbia Sheep blood agar (CA), Brucella sheep blood agar (BR) Laked Blood Kanamycin agar (LBK), 2 sets of Human Bi-layer Tween agar (HBT), and Rogosa agar. The CA and one set of HBT plates were incubated in 6% CO_2_ at 37°C for 48 hours and the BR, LBK, and Rogosa plates were incubated in an anaerobic chamber for 4–7 days. All lactobacilli isolated were tested for production of H_2_O_2_ in a qualitative assay on a tetra-methyl-benzidine agar plate. After 3 days of incubation in an anaerobic glove box at 37°C, the plates were exposed to ambient air for up to 30 minutes and observed for a blue color from the hydrogen peroxide horseradish reaction. The concentration of each microbe was assessed as colony forming units (cfu) per gram of vaginal fluid.

### Statistical Analysis

Data obtained for this substudy were analyzed based on all available data collected during the study period. There was no formal sample size calculation for this open label study. Assessment windows were defined as screening (Baseline level), Cycle 6 (targeted at Day 144; beginning of Cycle 6), and Cycle 13 (targeted at Day 358; 12 cycles plus 21 days of use during cycle 13). Data for participants with mistimed visits or early termination were included in the appropriate assessment window. If multiple observations fell within the same window, only results from the last assessment were used. Descriptive methods were used to summarize study participants’ characteristics, and for comparisons between participants who completed vs. those who discontinued the study early.

Changes in the detection of BV, VVC, and trichomoniasis in the substudy population as well as the Nugent score, vaginal pH and semi-quantitative cultures from baseline to either Cycle 6 or Cycle 13 were analyzed by McNemar’s χ^2^ test for dichotomous data (an exact version was used for comparisons with small sample sizes). A nonparametric test of marginal homogeneity was used for ordinal data. Wilcoxon signed-rank tests were employed to determine whether there were differences in the concentration for each of the microbes from baseline to follow up visits. A sensitivity analysis regarding changes in microflora overtime was conducted among subjects who completed all vaginal culture assessments at baseline, Cycle 6 and Cycle 13. The McNemar’s χ2 tests and Wilcoxon signed-rank tests were also used to compare the paired results of vaginal and ring cultures at the end of study.

A generalized linear mixed model–which accounts for the repeated measurements of the outcome variable over time–was fitted with the presence of a specific microorganism as the outcome and with duration of use of the NES/EE CVR as the primary predictor [0 = baseline (reference), 1 = Cycle6, 2 = Cycle13]. The outcome was recorded as a binary variable. The estimated effect of the NES/EE CVR on the presence of microorganisms was adjusted for the following covariates, which could be considered as potential confounders: demographic characteristics, i.e. age, marital status, history of recent urogenital tract infections obtained during screening, contraception history prior to entry, antibiotic use within 7 days prior to culture, the number of days between cyclic bleeding and the culture, sexual activity within three days prior to culture, ring expulsions and mean sexual intercourse per cycle.

The prevalence and 95% confidence interval of vaginal infections (BV, VVC, and trichomoniasis) and other urogenital infections as well as UTIs were calculated based upon all clinical trial reports of treatment emergent adverse events (TEAEs) that occurred among substudy participants, and among all participants from the Phase III trial, which included 14 other US sites. To account for repeated incidences of infections, a series of sensitivity analyses were conducted to calculate and compare the cumulative incidence rates by Poisson regression models between participants in the microbiology substudy and in the Phase III trial.

Data were analyzed using SAS statistical software 9.3 (Cary NC, USA). All analyses were two-tailed and P<0.05 was considered statistically significant.

## Results

### Participants’ Demographics and Disposition

Demographic characteristics of the substudy population are presented in Table 1. Most subjects were unmarried and had more than high school education. Overall 120 participants provided microbiological data at the Screening visits, 110 at Cycle 6 (median time: 145 days, range 28–250 days) and 61 at Cycle 13 (median time: 357 days, range 272–378 days). Fifty-two participants discontinued prematurely for a variety of reasons including 6 women who were discontinued due to the protocol change pertaining to BMI. Seven women completed the study according to protocol specifications, but did not complete a full 13 cycles due to late enrollment and expiration of the CVR (Fig 1). African-American women were more likely to terminate ring use prematurely (p = 0.007). There were no other baseline characteristics that were statistically different between completers and earlier discontinuers (all p values > 0.05).

**Table 1:** Demographic and baseline characteristics among substudy participants.

	Total participants (N = 120)		Study completers (N = 68)		Early discontinuers (N = 52)	
	n	(%)	n	(%)	n	(%)
**Age (**Mean ± SD)	24.5 ± 3.8		23.3 ± 3.6		24.1 ± 4.7	
Race*						
White	98	(81.7)	62	(91.2)	36	(69.2)
Black or African-American	16	(13.3)	4	(5.9)	12	(23.1)
Other/Unknown	6	(5.0)	2	(2.9)	4	(7.7)
**Ethnicity**						
Hispanic or Latina	7	(5.8)	3	(4.4)	4	(7.7)
Not Hispanic or Latina	113	(94.2)	65	(95.6)	48	(92.3)
**Marital status**						
Never married	104	(86.7)	57	(83.8)	47	(90.4)
Married	13	(10.8)	9	(13.2)	4	(7.7)
Divorced	3	(2.5)	2	(2.9)	1	(1.9)
**Education status**						
College degree or higher	62	(51.7)	37	(54.4)	25	(48.1)
Some college	50	(41.7)	27	(39.7)	23	(44.2)
High school diploma/equivalent	8	(6.7)	4	(5.9)	4.0	(7.7)
**Current Smoking**						
No	101	(84.2)	59	(86.8)	42	(80.8)
**Current Drinking**						
Yes	109	(90.8)	63	(92.7)	46	(88.5)
**Body Mass Index** (kg/m^2^ **) ……** [Mean ± SD]	23.1 ± 3.1		22.7 ± 2.6		23.7 ± 3.6	
**Gravidity**						
0	92	(76.7)	56	(82.4)	36	(69.2)
1	16	(13.3)	8	(11.8)	8	(15.4)
≥2	12	(10.0)	4	(5.9)	8	(15.4)
**Parity**						
0	105	(87.5)	61	(89.7)	44	(84.6)
≥1	15	(12.5)	7	(10.3)	8	(15.4)
**Desire to have children after the study**						
Yes	92	(76.7)	53	(77.9)	39	(75.0)
Contraceptive methods prior to enrollment)**						
Vaginal ring (NuvaRing)	54	(40.3)	32	(41.0)	22	(39.3)
Combined oral contraceptives	22	(16.4)	12	(15.4)	10	(17.9)
Condoms	40	(30.0)	23	(29.5)	17	(30.4)
Withdrawal	6	(4.5)	3	(3.9)	3	(5.4)
Other***	12	(9.0)	8	(10.3)	4	(7.1)

*p<0.05

**Multiple choices allowed per subject. Seven subjects did not respond.

*** Other includes abstinence, rhythm method, and transdermal patch.

### Changes in the Detection of Vaginal Infections and the Microbiota

**Fig 2:**
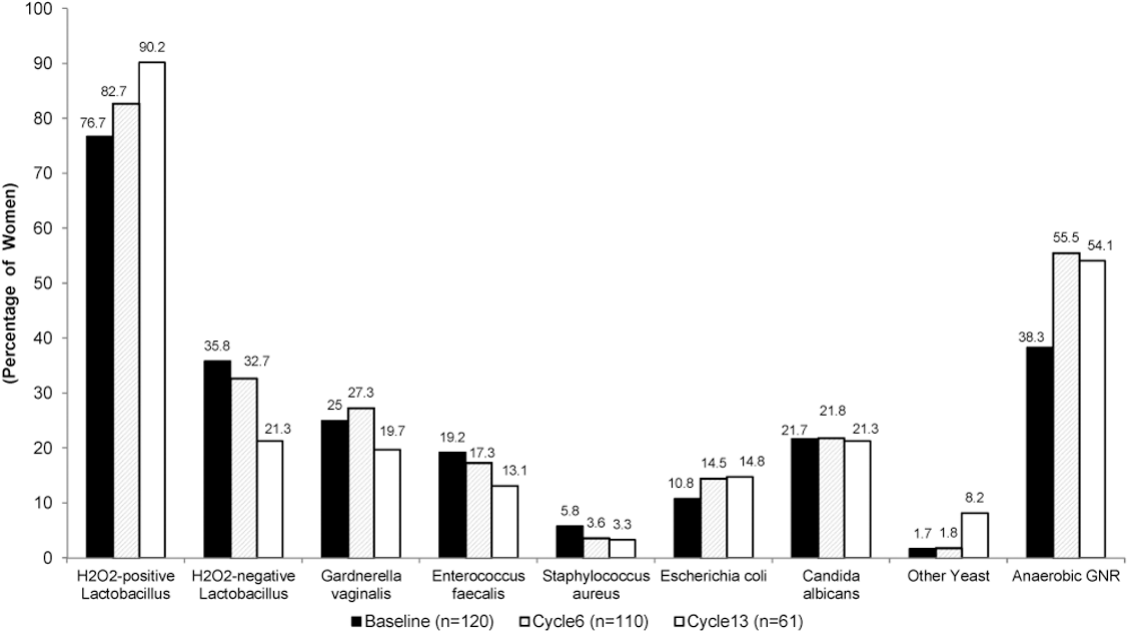
Prevalence of selected vaginal microbiota by assessment window. Visit timing: Cycle 6 (targeted at Day 144 [beginning of Cycle 6], range 2 to 251); Cycle 13 (targeted at Day 358 [12 cycles plus 21 days of use during cycle 13], range 252 to 358).

There were no significant changes in the detection rate of BV between baseline to Cycle 6 or from baseline to Cycle 13 based on Amsel’s criteria or Nugent scores (Table 2). Similarly, there were no significant changes in the detection of VVC over the study period, and no cases of trichomoniasis were identified during three assessments periods (one case of trichomoniasis was found at another time point during the study and was documented as an AE and noted in Fig 2). In addition, no significant change was observed regarding the number of vaginal neutrophils on the Gram-stained vaginal smear, or in the percentage of women with vaginal pH > 4.5.

**Table 2:** Change in vaginal infections and selected vaginal microbiota by assessment visit and the comparison of vaginal culture and ring culture.

	Baseline to Cycle 6(N = 110)			Baseline to Cycle13(N = 61)			Vaginal culture vs. Ring culture**(N = 72)		
	Baselinen (%)	Cycle 6n (%)	Concordant pairs n (%)	Baselinen (%)	Cycle 13n (%)	Concordant pairs n (%)	Vaginal culturen (%)	Ring culturen (%)	Concordant pairsn (%)
BV (Amsel criteria)	1 (0.9)	3 (2.7)	106 (96.4)	0 (0.0)	1 (1.6)	60 (98.4)			
Yeast Vaginitis	2 (1.8)	3 (2.7)	105 (95.5)	1 (1.6)	3 (4.9)	57 (93.4)			
Nugent Score > = 7^#^	10 (9.3)	12 (11.1)	100 (92.6)	1 (1.6)	5 (8.3)	56 (80.0)			
Neutrophil counts 2+/3+	10 (9.1)	17 (15.5)	91 (81.6)	6 (9.8)	10 (16.3)	47 (77.1)			
Vaginal pH > 4.5	16 (23.6)	26 (23.6)	80 (72.7)	11 (18.3)	11 (18.3)	42 (70.0)			
H_2_O_2_-positive *Lactobacillus*	85 (77.2)	91 (82.7)	86 (78.2)	49 (80.3)	55 (90.2)	48 (78.7)	65 (90.3)	66 (91.7)	67 (93.1)
H_2_O_2_-negative *Lactobacillus*	41 (37.2)	36 (32.7)	65 (59.1)	25 (41.0)	13 (21.3)*	36 (63.9)	16 (22.2)	12 (16.7)	66 (91.7)
*Gardnerella vaginalis*	25 (22.7)	30 (27.3)	95 (86.4)	13 (21.3)	12 (19.7)	51 (83.6)	16 (22.2)	16 (22.2)	68 (94.4)
*Enterococcus faecalis*	21 (19.1)	20 (17.3)	88 (80.0)	7 (13.1)	8 (13.1)	54 (88.5)	12 (16.7)	17 (23.6)	59 (81.9)
*Staphylococcus aureus*	7 (6.3)	4 (3.6)	101 (91.8)	3 (4.9)	2 (3.3)	56 (91.8)	4 (5.6)	3 (4.2)	67 (93.1)
*Escherichia coli*	12 (10.9)	16 (14.5)	88 (80.0)	6 (9.8)	9 (14.8)	47 (77.1)	9 (12.5)	6 (8.3)	65 (90.3)
*Candida albicans*	24 (21.8)	24 (21.8)	86 (78.2)	13 (21.3)	13 (21.3)	44 (72.1)	15 (20.8)	18 (25.0)	69 (95.8)
Other Yeast	2 (1.8)	2 (1.8)	108 (98.2)	1 (1.6)	5 (8.2)	60 (98.4)	5 (6.9)	4 (5.6)	71 (98.6)
Anaerobic GNR	39 (35.4)	61 (55.5)*	70 (63.6)	21 (34.4)	33 (54.1)	37 (60.7)*	40 (55.6)*	26 (36.1)	56 (77.8)

Visit timing: Cycle 6 (targeted at Day 144 [beginning of Cycle 6], range 2 to 251); Cycle 13 (targeted at Day 358 [12 cycles plus 21 days of use during cycle 13], range 252 to 358).

# One subject without Nugent Score data at screening.

* McNemar’s tests: p<0.05.

** Performed at study exit (Cycle 13 [n = 58] or early discontinuation visit [n = 14]).

**Fig 3:**
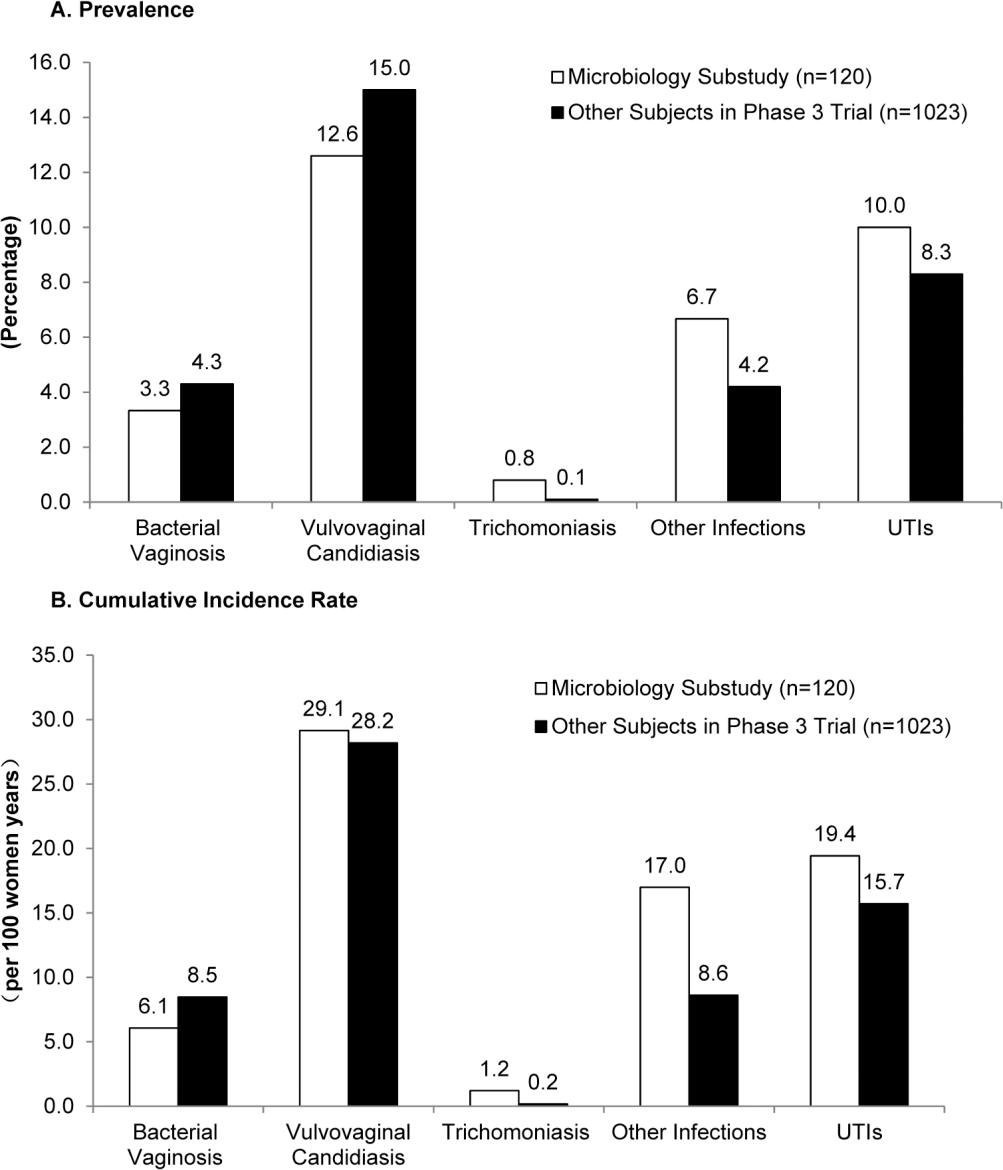
Prevalence and cumulative incidence rates of vaginal infections and urogenital infections in women in the substudy and remainder of women in Phase III trial.

The prevalence of selected components of the vaginal microbiota at baseline and follow up visits are presented in Fig 3. The proportion of women colonized by H_2_O_2_ positive *Lactobacillus* increased from 76.7% at baseline to 82.7% at Cycle 6 and to 90.2% at Cycle 13. Among 61 study participants who completed all three assessments of the vaginal microbiota, the proportion difference between baseline and cycle 13 was 80.3% and 90.2%) (p = 0.07, Table 2). The median concentration of H_2_O_2_-positive *Lactobacillus* remained high, 10^7^ cfu/gm at baseline, Cycle 6 and Cycle 13 (Table 3). We found no statistically significant increase in the frequency or the concentration of H_2_O_2_ producing *Lactobacillus*. The prevalence of H_2_O_2_-negative *Lactobacillus* remained at the same level from baseline to Cycle 6, but both the prevalence and the concentration decreased significantly–from baseline to Cycle 13 (Tables 2 and 3, p = 0.02 and 0.008, respectively). Most participants did not have positive cultures or they had very low levels of *Gardnerella vaginalis*, *Enterococcus faecalis*, *Staphylococcus aureus*, *Escherichia coli*, *Candida albicans* or other yeast during any of their study visits, and the distribution and median concentration of these organisms did not vary from baseline to the follow up (Tables 2 and 3). Anaerobic GNRs increased significantly from baseline to Cycle 6, (35% to 55%, p<0.01) and between baseline and Cycle13 (34% to 54.1%, p = 0.04). The median concentration of anaerobic GNRs, however, remained at low levels (10^4^ cfu/gm at baseline, 10^3^ cfu/gm at Cycles 6 and 13; Table 3) so the modest increase in the frequency of this group of microorganisms is of doubtful clinical significance.

**Table 3:** Median concentration (cfu/gm) of selected vaginal microbiota at each assessment window and for ring culture among positive cultures.

	Each Assessment visit			Vaginal culture vs. Ring culture ^c^	
	Screening(N = 120)	Cycle6(N = 110)	Cycle13(N = 61)	Vaginal (N = 72)	Ring (N = 72)
H_2_O_2_-positive *Lactobacillus n* ^*a*^ Median [IQR]^b^	92 10^7^ [10^6^, 10^8^]	91 10^7^ [10^7^, 10^8^]	55 10^7^ [10^7^,10^8^]	65 10^7^ [10^7^, 10^8^]	66 10^7^ [10^6^, 10^8^]
H_2_O_2_-negative *Lactobacillus n* ^*a*^ Median [IQR]^b^	43 10^7^ [10^6^, 10^8^]	36 10^7^ [10^6^, 10^7^]	13 10^7^ [10^6^, 10^8^]	16 10^7^ [10^6^, 10^7^]	12 10^6^ [10^6^, 10^7^]
*Gardnerella vaginalis n* ^*a*^ Median [IQR]^b^	30 10^7^ [10^6^, 10^7^]	30 10^7^ [10^6^, 10^8^]	12 10^7^ [10^6^, 10^7^]	16 10^7^ [10^6^, 10^8^]	16 10^7^ [10^6^, 10^8^]
*Enterococcus faecalis n* ^*a*^ Median [IQR]^b^	23 10^3^ [10^3^, 10^4^]	20 10^3^ [10^2^, 10^4^]	8 10^3^ [10^2^, 10^7^]	12 10^3^ [10^2^, 10^4^]	17 10^3^ [10^2^, 10^4^]
*Staphylococcus aureus n* ^*a*^ Median [IQR]^b^	7 10^3^ [10^2^, 10^4^]	4 10^3^ [10^3^, 10^4^]	2 10^3^ [10^3^, 10^3^]	4 10^3^ [10^3^, 10^4^]	3 10^3^ [10^3^, 10^5^]
*Escherichia coli n* ^*a*^ Median [IQR]^b^	13 10^3^ [10^2^, 10^3^]	16 10^3^ [10^2^, 10^5^]	9 10^3^ [10^2^, 10^5^]	9 10^3^ [10^2^, 10^5^]	6 10^3^ [10^2^, 10^4^]
*Candida albicans n* ^*a*^ Median [IQR]^b^	26 10^3^ [10^3^, 10^6^]	24 10^3^ [10^3^, 10^5^]	13 10^4^ [10^3^, 10^5^]	15 10^4^ [10^3^, 10^5^]	18 10^4^ [10^3^, 10^5^]
Other Yeast *n* ^*a*^Median [IQR]^b^	2 10^4^ [10^2^, 10^5^]	2 10^6^ [10^2^, 10^6^]	5 10^3^ [10^4^, 10^6^]	5 10^6^ [10^4^,10^6^]	4 10^7^ [10^4^, 10^7^]
Anaerobic GNR *n* ^*a*^ Median [IQR]^b^	46 10^4^ [10^2^, 10^6^]	61 10^3^ [10^2^, 10^5^]	33 10^3^ [10^2^, 10^3^]	40 10^3^ [10^2^, 10^5^]	26 10^3^ [10^2^, 10^5^]

Visit timing: Cycle 6 (targeted at Day 144 [beginning of Cycle 6], range 2 to 251); Cycle 13 (targeted at Day 358 [12 cycles plus 21 days of use during cycle 13], range 252 to 358).

^a^ Number of subjects with positive culture results at each assessment for each specific microorganism that was cultured.

^b^ cfu/gm: colony forming units (cfu) per gram.

^c^ Performed at study exit visit (Cycle 13 [n = 58] or early discontinuation visit [n = 14]).

The generalized linear mixed model revealed that other significant factors were associated with colonization of anaerobic GNRs among ring users. These included younger age (OR 0.89, 95% CL 0.81–0.99), mean sexual intercourse frequency per cycle greater than 8 (compared to those who had sexual intercourse 4 times or less per cycle: OR 3.42, 95% CL 1.35–8.67), and having the culture swab obtained within three days after vaginal intercourse (OR 2.26, 95% CL 1.09–4.70). Colonization of specific organisms at baseline was generally associated with colonization with the same microbes during CVR use, specifically, H_2_O_2_ positive *Lactobacillus*, anaerobic GNRs, *Gardnerella vaginalis*, *Enterococcus faecalis* and *Candida albicans* (each p < 0.01).

### Prevalence of Vaginal Infections

During CVR use, 4 participants in the substudy (3.3%, 95% CI: 0.9~8.3%) were clinically diagnosed with BV, 18 (15.0%, 95% CI: 7.2~20.0%) with VVC, and 1 (0.8%, 95%CI: 0.02~4.5%) with trichomoniasis. These were documented as TEAEs, as were other diagnosed urogenital infections including chlamydial cervicitis (3), genital herpes episodes (3), anogenital warts (1), gonococcal cervicitis (1) and HPV (1). Twelve (10%, 95% CI: 5.3~16.8%) women were diagnosed with UTIs. The prevalence of clinically diagnosed vaginal infections, other vulvo-vaginal infections and UTIs among women in the microbiology substudy were comparable to the rates calculated for the remaining Phase III participants (n = 1023) (all p > 0.05, Fig 2).

Four women in the substudy had repeated VVC, one had repeated BV, and one had repeated chlamydial cervicitis. Two women had repeated herpes simplex virus reactivation, and 3 had recurrent UTIs. The cumulative incidence rates for BV, VVC, trichomoniasis, and UTI were 6.1 (95%CI: 2.5~14.6), 29.1(95%CI: 19.5~43.5), 1.2(95%CI:0.2~8.6), and 19.4 (95%CI: 11.9~31.7) per 100 women years, respectively, which were comparable to the rates among women from the other Phase III clinical trial sites (all p > 0.05, Fig 2). The cumulative incidence rate of other VVC infections among women in the substudy was significantly higher than the rate for the remainder of participants in the Phase III trial (17.0, 95% CI: 10.1~28.7 vs. 8.6, 95%CI: 6.6~11.2) per 100 women year, p = 0.02, Fig 2). There were no cases of pelvic inflammatory disease (PID) in the microbiology substudy or in the overall Phase III study.

### Comparison of Vaginal and Ring Cultures

Seventy two (72) of the 120 women in this microbiology study had their rings collected and cultured at study exit. The remaining 48 women did not have ring cultures performed, because follow-up vaginal cultures were not performed (n = 10), they were lost-to-follow-up (n = 6), experienced ring expulsion (n = 1), or removed their ring prior to the study visit (n = 31). A comparison of corresponding ring and vaginal cultures in the 72 women with such results is presented in Table 2. Generally, there was a high level of agreement (concordance >90% for H_2_O_2_ positive or negative *Lactobacillus*, *Gardnerella vaginalis*, *Staphylococcus aureus*, *E*.*coli*, *Candida albicans*, and other yeast; >80% for *Enterococcus*) between the vaginal cultures and the cultures obtained from the CVR surface. There was an exception with the anaerobic GNRs, which were significantly more likely to be present in the vaginal fluid compared to the CVR (p = 0.0005). We found no significant difference between CVR and vaginal fluid cultures for the remainder of the cultured organisms. *Staphylococcus aureus* was rare in both the vagina and on the CVR surface.

## Discussion

In this study, we examined changes in selected components of the vaginal microbiota and the risk of vaginal infections associated with cyclic use of the same NES/EE CVR for up to one year. This study was conducted among women from one clinical site who were also participating in a large Phase III clinical trial of the NES/EE CVR. We found no substantial effects of long term repeated use of the NES/EE CVR up to 13 cycles on the vaginal ecosystem, and no significant change on the incidence of vaginal infections, specifically VVC, BV, or *Trichomonas vaginalis*.

Overall, 75% of reproductive aged women will have at least one episode of VVC in their lives and 40% to 50% of them will have a second episode [19]. These occurrences have been linked to female hormonal fluctuations [19]. Estrogen promotes a glycogen-rich vaginal environment in which *Candida* or other yeast species can thrive and give rise to symptomatic infections. Among the women in this study, the prevalence and cumulative incidence of VVC were 15% and 29.1 per women-years, respectively, which was comparable to the rates in the larger Phase III clinical trial. *Candida albicans* colonization was observed among 21% of the NES/EE CVR users in the substudy, which is comparable to the overall prevalence of this yeast infection among women of reproductive age [20].

BV is another common reproductive tract infection among women of childbearing age and is associated with factors such as race, young age, marital status, douching, frequent sexual intercourse, numbers of sexual partners [7, 21, 22]. Use of hormonal contraceptives (oral, injections, or implants) has been found to be protective against the occurrence of BV [21], and there has been no evidence that the use of a combined hormonal CVR increases the risk of BV acquisition [12–14, 22–24]. Likewise, in this study, we found no significant change in the occurrence of BV from baseline up to one year of ring use, which is consistent with the high concentration of H_2_O_2_ positive *Lactobacillus* among participants in this microbiology study who were cultured following up to one year of use of the NES/EE CVR. Additionally, the prevalence and cumulative incidence rate of BV among women at the substudy site and the remaining pivotal Phase III trial sites was comparable to the rates reported for women in other studies based on Amsel criteria [25].

In the normal vaginal environment, lactobacilli protect against overgrowth of endogenous anaerobic bacteria by producing hydrogen peroxide, lactic acid, and bacteriocins [20]. Results of a study with monthly NuvaRing users revealed that these women had a higher concentration of H_2_O_2_ positive *Lactobacillus* when compared with the oral contraceptive users [15]. In our study, H_2_O_2_ positive *Lactobacillus* dominated the vaginal environment as evidenced by the high median concentration (10^7^cfu/gm) among NES/EE CVR users and the elevated prevalence from approximately 77% at baseline to 83% and 90% at Cycles 6 and 13, respectively. It has been speculated that increased estrogen may facilitate lactic acid production by increasing levels of available glycogen in epithelial cells, thereby inhibiting BV-associated organisms [21, 22, 26]. Such exposure may stem from vaginally-delivered contraceptives that contain estrogen and can result in preferential delivery of hormones to the genital tissues [27].

In this microbiology substudy, the one vaginal flora alteration observed was a statistically significant but clinically insignificant elevated prevalence of anaerobic GNRs. Although the change in occurrence of anaerobic GNRs has been recognized as one of the BV-associated microorganisms and may be associated with development of pelvic inflammatory disease [28], the median concentration of anaerobic GNRs among subjects in this study was very low (10,000 cfus/gm) during all follow-up visits in comparison to the H_2_O_2_ positive *Lactobacillus* that were present at 10,000 fold higher concentrations. Women harboring low concentrations of anaerobic microorganisms are usually asymptomatic [20]. Similar to findings reported in the literature, the positive culture of anaerobic GNRs at screening and during NES/EE CVR use was associated with younger age, frequent acts of sexual intercourse (> 8 times per cycle) and having the vaginal culture done within 3 days after sexual intercourse. This latter finding is consistent with the fact that seminal fluid has a neutral to alkaline pH (7.2–8.0) and contains enzymes that inactivate hydrogen peroxide, which in turn may change (raise) the vaginal pH and increase the likelihood of anaerobic bacterial overgrowth [26].

Importantly, we found a high level of agreement between the NES/EE CVR and the vaginal fluid cultures in this microbiology substudy. This result suggests that the ring surface does not promote the proliferation of microorganisms, including *Staphylococcus aureus*, which has been implicated as a causative factor of toxic shock syndrome [29]. *Staphylococcus aureus* was rarely cultured either from the vagina or from the CVRs used by participants in this study. The only difference observed between CVR and vaginal culture was related to colonization with anaerobic GNRs. Significantly more subjects had anaerobic *GNRs* cultured from the vagina compared with results from the ring. This finding may be due to the fact that unlike other fastidious anaerobic organisms, i.e. *Gardnerella vaginalis*, *Staphylococcus aureus*, *Enterococcus faecalis*, *and Escherichia coli*, anaerobic GNRs are more sensitive to both oxygen exposure and temperature variations, which could have been factors associated with ring removal and transport procedures and resulted in a lower prevalence of Anaerobic GNRs from the ring cultures [30].

The strength of this study was its prospective design to evaluate the effects of one year use of the same CVR on the vaginal ecosystem and the incidence of vaginal infections, specifically BV, VVC, and trichomoniasis during cyclic use of a single NES/EE CVR up to one year. It also included assessments of more microorganisms compared to previous studies [18, 19], and provided additional information about the effects of CVR use on the vaginal micro flora and microorganisms cultured from the rings at the end of the study, which had not been described in previous reports.

Study limitations included the lack of a comparison group of women who were not using the CVR, use of a single study site, and a high dropout rate among participants at the study site. In general, the vaginal infection rate at this single center was similar to that found for the larger Phase III population supporting the evidence from this single center. Although the dropout rate was high, the percentage of women diagnosed with BV, VVC, trichomoniasis and other urogenital infections and UTIs among the women who discontinued was comparable to the rates calculated for women who completed the study (S1 Fig). Furthermore, the culture results for women who completed all 3 evaluations during one year of use were similar to results obtained from all women enrolled in this safety study. While the demographic characteristics of participants in this study (i.e. race and ethnicity) were representative of the US population [31], the rate of discontinuation was higher among African-American women compared to White women. Hence, the overall findings may not be totally representative of African-American women. Similarly, the young age and exclusion of women with BMIs > 29.0, may not have been reflective of other populations in the US or internationally, and vaginal infection rates vary among these populations [7, 20, 21]. Future studies that include women from more diverse populations, specifically from countries in sub-Saharan Africa and south Asia where the unmet need for contraception remains high and vaginal infections are prevalent [1, 2, 24] should be considered. It should be noted that for this microbiology study, we used direct microscopic observation (wet mount) of vaginal secretions to diagnose *Trichomonas* vaginalis. Although this technique is rapid, inexpensive, and most commonly used in practice settings, its sensitivity is generally 44 to 68% [32], and we may have underestimated the occurrence of trichomonasis in this study (no cases occurred during three planned assessments and only one case was reported as an adverse event over one year of use). The recent development of nucleic acid amplification tests for the detection of *Trichomonas* vaginalis may be useful for future investigations [32]. The culture-based methods used in this study did not include culture independent methods such as pyrosequencing that may have revealed novel microorganisms that are not detected with traditional cultivation based methods [33]. However, it is also recognized that such contemporary techniques (e.g. metagenomics) underestimate microbial diversity because they fail to detect minor populations present at low abundance while traditional culturing is more sensitive for detection of species with low abundance that may play a role in disease [34]. Srinivasan et al [35] investigated the bacterial communities in women with BV and found that pyrosequencing techniques failed to detect pathogens of importance including Group B *Streptococcus*, *Staphylococcus aureus*, *E coli* and *Candida albicans*. Finally, we have observed that other studies of vaginal rings have included assessment of biofilms composed of epithelial cells and microorganisms. In one such study, vaginal rings were evaluated for evaluation of biofilm using scanning electron microscopy following one month of use [36]. Not surprisingly, all of the used vaginal rings showed evidence of biofilm but the amount of biofilm was unrelated to whether or not women had evidence of BV. Similarly, Miller et al. [37] examined a NuvaRing worn for 28 days by a healthy female volunteer and did not observe embedded bacteria, erosion or structural changes compared to an unused ring. While further study of this may be of interest, results from this study suggest that the possible presence of biofilm on the NES/EE CVR used cyclically for up to one year did not impact negatively on vaginal safety.

## Conclusions

The results of this study support the safety of the long acting NES/EE CVR with respect to its effects on the vaginal microbiota and the incidence of vaginal infections. Findings from this study are important for further exploration of effects of vaginal rings on the vaginal microbiome including other rings in development for delivery of contraceptives, microbicides to prevent transmission of STIs including HIV and HPV, and for multipurpose prevention technologies that are designed to protect against both STIs and pregnancy [2, 3].

## Supporting Information

S1 Checklist TREND Checklist. (DOCX)

S1 Dataset Microbiology substudy demographics.xpt. Microbiology substudy disposition.xpt. Microbiology substudy examination.xpt. Microbiology sub study vital signs.xpt. Microbiology and main study vaginal AEs.xpt.
(ZIP)

S1 Fig Percentage of Vaginal Infections and Urogenital Infections in Women who discontinued from study and those who completed study. (TIF)

S1 Protocol Microbiology Substudy Protocol. (PDF)
