# Supplementary figures and images for: Effects of a One Year Reusable Contraceptive Vaginal Ring on Vaginal Microflora and the Risk of Vaginal Infection: An Open-Label Prospective Evaluation
Source: PLoS One. 2015 Aug 12;10(8):e0134460. doi: 10.1371/journal.pone.0134460 (PMC4534458; doi:10.1371/journal.pone.0134460)

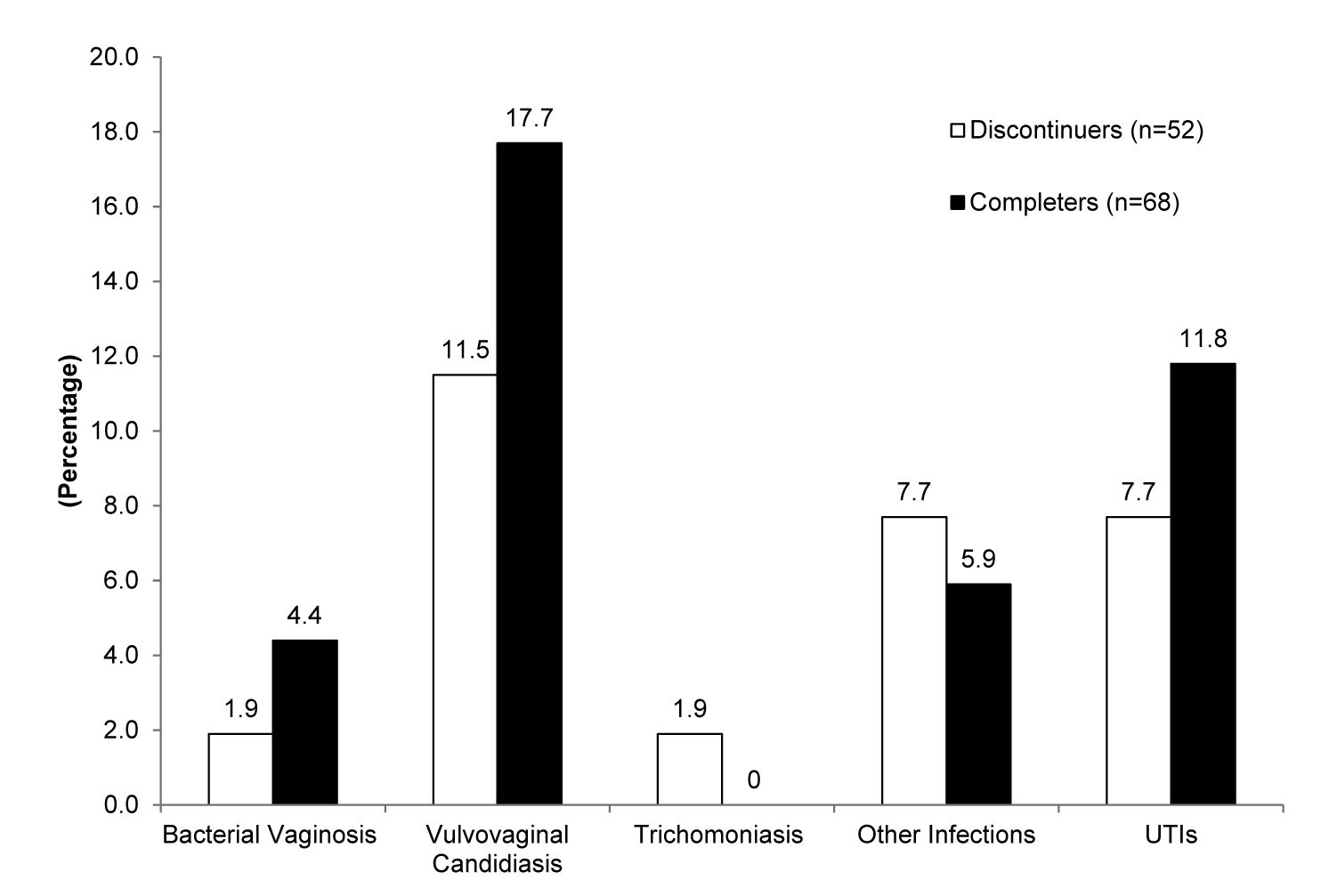

Supplement: S1 Fig — (TIF) [file pone.0134460.s003.tif]
