# Supplementary material for: Effects of a One Year Reusable Contraceptive Vaginal Ring on Vaginal Microflora and the Risk of Vaginal Infection: An Open-Label Prospective Evaluation
Source: PLoS One. 2015 Aug 12;10(8):e0134460. doi: 10.1371/journal.pone.0134460 (PMC4534458; doi:10.1371/journal.pone.0134460)
Supplement: S1 Protocol — (PDF) [file pone.0134460.s004.pdf]

# APPENDIX 8

## A multicenter, open-label study on the efficacy, cycle control and safety of a contraceptive vaginal ring delivering a daily dose of 150 μg of Nestorone® and 15 μg of ethinyl estradiol (150/15 NES/EE CVR)

## MICROBIOLOGY SUBSTUDY

**INTRODUCTION AND BACKGROUND:** Over the past decade, the importance of an intact vaginal ecosystem in resistance to urogenital infection has been highlighted. Most of the published data describing the effects of vaginal products on the vaginal microbial ecosystem have assessed short-term exposures. Only one study has been published demonstrating the effects of routine use of a vaginal product on microflora (1). This study evaluated the impact of the long-term use of five different formulations of N-9 on vaginal flora in sexually active women in mutually monogamous relationships. The investigators found the changes in prevalence of vaginal microbes after N-9 use were minimal, regardless of N-9 formulation. However, when both N-9 concentration and number product uses were taken into account, N-9 did have dose-dependent effects on the increased prevalence of anaerobic gram-negative rods (OR=2.4 [95% CI 1.1-5.3]), $H_2O_2$-negative lactobacilli (OR= 2.0 [95% CI 1.0-4.1]), and bacterial vaginosis (OR= 2.3 [95% CI 1.1-4.7]).

Such findings are important because women with vaginal colonization by lactobacilli are less likely to have (2) and acquire (3) sexually transmitted infections such as Herpes simplex virus 2 (4) and human papilloma virus (5), and are also less likely to acquire bacterial vaginosis (3). Two cross-sectional studies and one longitudinal study have shown an association between the presence of vaginal lactobacilli and decreased prevalence or incidence of HIV (6-8).

Accordingly, understanding the effects of a vaginal product on the microflora is important as such changes can have a significant public health impact. Therefore, this study will include appropriate evaluations at baseline, after 6 cycles of use (visit 3), and after 13 cycles of use (visit 5). These evaluations will be performed during scheduled visits and will have minimal impact on the participants.

The microbiology sub study will enroll 100 subjects at a single center to evaluate changes in vaginal flora associated with use of the same CVR for one year. At visit 5, when the study ring is removed, quantitative cultures of the vaginal ring will also be performed.

**SUBSTUDY OBJECTIVE:** To evaluate if the presence of a contraceptive vaginal ring containing Nestorone® and ethinyl estradiol that is used three weeks out of four and reused cyclically for 13 cycles increases the risk of infection.

## RATIONALE, BENEFITS AND RISKS:

This sub study is being performed to address additional safety questions pertaining to cyclical use of a single ring for 13 cycles and to determine if periodic insertion of the same vaginal ring changes the microflora of the vagina and thereby predisposes women to vaginal infections. The benefit will be the additional information obtained to inform clinicians and women about any possible risk of infection related to cyclical use of the same CVR over one year. This study will involve a vaginal smear and aspiration of vaginal fluid taken 3 times during the course of the study and as part of the pelvic exam that must be done in accordance with procedures for the core study. Taking such a smear does not generally entail any additional risks beyond what is normally experienced by women having a pelvic exam, which can involve some mild discomfort during the exam itself.

## CLINICAL STUDY PROCEDURES:

- *For all study participants*, CBC results, including differential, will be examined at baseline, Visit 3 and Visit 5.
- *For the sub study participants*, the following evaluations will be performed at the time of the vaginal examination during the screening visit, Visit 3 and Visit 5 (see flow chart at end of this section):
    - Quantitative vaginal cultures for anaerobic gram-negative rods, *E. coli*, other gram-negative rods, Lactobacillus ($H_2O_2$ positive and negative strains will both be identified), *Candida albicans*, other yeast, *Enterococcus*, *Gardnerella vaginalis,* and *Staphylococcus aureus*.
    - Vaginal wet (saline) prep and KOH prep to evaluate for bacterial vaginosis (Amsel's criteria) and white blood cells
        - Amsel's criteria (9). The presence of three of the four following criteria gives the diagnosis of bacterial vaginosis:
            1. A homogenous excessive vaginal discharge;
            2. An alkaline vaginal pH;
            3. A positive whiff test when a drop of vaginal fluid is added to a 10% potassium hydroxide solution; and
            4. The presence of 20% or more clue cells when a microscopic examination of the saline hanging drops preparation is done.
        - white blood cells quantification:
            1. Absent
            2. Few
            3. Moderate
            4. Many
    - Vaginal KOH prep to evaluate for yeast (spores/hyphae)
    - Vaginal smear for bacterial vaginosis evaluation (Nugent score)
        - a larger number of women will be diagnosed with bacterial vaginosis using the Nugent score as compared to the Amsel criteria; this study will record both measures.
        - Nugent score: A gram stain from a vaginal smear interpreted by a standard method for the diagnosis of bacterial vaginosis (10).
            1. Score 0 to 3 = normal, Lactobacillus-predominant flora

2. Score 4 to 6 = intermediate flora
3. Score 7 to 10 = bacterial vaginosis

- body temperature

Microbiology Sub Study Flowchart

| Cycle | Screening | 1 | 3 | 6 | 9 | 13 | 14 | Follow-up |
|---|---|---|---|---|---|---|---|---|
| Visit | 0 | 1 | 2 | 3 | 4 | 5 | 6 | 7.1-7.3 |
| Temperature | X | | | X | | X | | |
| Vaginal smear (wet mount/saline preparation) | X | | | X | | X | | |
| KOH analysis | X | | | X | | X | | |
| Vaginal pH | X | | | X | | X | | |
| Whiff test | X | | | X | | X | | |
| Gram stain | X | | | X | | X | | |
| Vaginal culture | X | | | X | | X | | |

- *For the sub study participants*, the vaginal ring will be removed at Visit 5 by the study clinician using sterile gloves and cultured.

**LABORATORY PROCEDURES:**

**Specimens**

1. Two vaginal swabs in Port-a-cul tube for quantitative culture (Visits: screen, 3, and 5)
2. The CVR will be placed in an appropriate sterile transport container (Visit 5)
3. Vaginal smear for Gram stain (Visits: screen, 3, and 5)

**Specimen rejection**

Testing will not be done on specimens > 96 hours old, specimens with no subject identifier, and frozen samples.

**Specimen processing**

Vaginal smears: Gram stain and record the semi-quantitative number of WBC's and score for BV by the Nugent criteria on the report form.

Vaginal swabs in Port-a-cul tube and Amies transporter: Transfer to biological safety hood for inoculation of media.

Vaginal ring: Transfer to biological safety hood for inoculation of media.

**For quantitative vaginal culture (Port-a-cul tube)**
All specimens are processed using universal safety precautions, wearing gloves, gown and working in a laminar flow biological safety hood. All media to be inoculated are labeled with the study name, subject identifier and the date.

The vaginal swabs are setup for quantitative culture as follows:
1. Remove the 2 swabs from the Port-a-Cul and place them into 1.5-mL of pre-modified Hank's solution and vortex.
2. Remove the inoculated salt solution with a pipette and transfer it to a small sterile tube (snap cap).
3. Make serial 1:10 dilutions from $10^{-1}$ to $10^{-7}$ in sterile pre-reduced saline. (The original suspension is $10^{-1}$ and the plate that it is inoculated with is $10^{-2}$.)
4. Use 100-ul of the original suspension and of each of the dilutions to inoculate the following media: $10^{-2}$ to $10^{-7}$ on Columbia Sheep blood agar (BA) and Brucella sheep blood agar (BR), $10^{-2}$, $10^{-3}$, $10^{-5}$, and $10^{-7}$ on Laked Blood Kanamycin agar (LBK) and 2 sets of Human Bi-layer Tween agar (HBT) , and $10^{-2}$ on Rogosa agar.
5. Use a sterile dally rod to spread the inoculum on the plates with dilutions from $10^{-3}$ and up. The same dally rod can be used for all plates with the same dilution and one dilution lower. Always spread the plates with the higher dilution first. The plates with $10^{-2}$ dilution should be streaked according to the procedure for semi-quantitative cultures (4 quadrants).

The Columbia agar and one set of HBT plates are incubated in 6%$CO_2$, 37oC for 48 hours and the Brucella agar, LBK, and Rogosa plates are incubated in an anaerobic chamber for 4-7 days.

**For quantitative culture of the CVR (in appropriate transport container)**
All specimens are processed using universal safety precautions, wearing gloves, gown and working in a laminar flow biological safety hood. All media to be inoculated are labeled with the study name, subject identifier and the date.
The setup for quantitative culture will be as follows:
1. A 1.0-mL volume of modified Hank's solution will be added to the transport container and vortexed.
2. Remove the inoculated salt solution with a pipette and transfer it to a small sterile tube (snap cap).
3. Make serial 1:10 dilutions from $10^{-1}$ to $10^{-7}$ in sterile pre-reduced saline. (The original suspension is $10^{-1}$ and the plate that it is inoculated with is $10^{-2}$).
4. Use 100-ul of the original suspension and of each of the dilutions to inoculate the following media: $10^{-2}$ to $10^{-7}$ on Columbia Sheep blood agar (BA) and Brucella sheep blood agar (BR), $10^{-2}$, $10^{-3}$, $10^{-5}$, and $10^{-7}$ on Laked Blood Kanamycin agar (LBK) and 2 sets of Human Bi-layer Tween agar (HBT) , and $10^{-2}$ on Rogosa agar.
5. Use a sterile dally rod to spread the inoculum on the plates with dilutions from $10^{-3}$ and up. The same dally rod can be used for all plates with the same dilution and one dilution lower. Always spread the plates with the higher dilution first. The plates with $10^{-2}$ dilution are streaked according to the procedure for semi-quantitative cultures (4 quadrants).

The Columbia agar and one set of HBT plates are incubated in 6%$CO_2$, 37oC for 48 hours and the Brucella agar, LBK, and Rogosa plates are incubated in an anaerobic chamber for 4-7 days.

**Identification**

The following organisms only will be identified:

- *Lactobacillus*, test for $H_2O_2$
- *Gardnerella vaginalis*
- *Enterococcus*
- *Candida albicans*
- Yeast, other
- *Staphylococcus aureus*
- *E. coli*
- Other Gram negative rods
- Anaerobic Gram negative rods

**Reporting**

Gram stains and culture results are recorded on the laboratory reporting form.. The site will transcribe these results to the CRF, which is transmitted to HD.. Both the laboratory reporting form and CRF must be signed. The original laboratory reporting form and CRF are kept on file with other records for the CCN006 protocol as outlined for Investigative Record Management.

**DATA ANALYSIS:**

Data obtained from women participating in this substudy will be assessed and compared before, during (at the sixth cycle of CVR use) and after 13 cycles of treatment. This analysis will include a comparison of body temperature, and WBC results over time. It will also include a comparison of results of cultures taken for the presence of anaerobic bacteria and smears taken to evaluate for the presence of bacterial vaginosis. Nugent scores and all other quantitative variables will be analyzed by repeat measure ANOVA in the set of women who complete the study. WBC and body temperature results will be closely examined for those women who have positive culture and/or smear results.

The presence and/or absence of organisms found on CVRs following removal will be described.

**REFERENCES:**

1. Schreiber CA, Meyn L, Creinin MD, Barnhart KT, Hillier SL. The effects of long-term use of nonoxynol-9 on vaginal flora. Obstet Gynecol 2006;107:136-43.
2. Hillier SL, Krohn MA, Nugent RP, Gibbs RS. Characteristics of three vaginal flora patterns assessed by gram stain among pregnant women. Am J Obstet Gynecol 1992;166:938-44.
3. Hawes SE, Hillier SL, Benedetti J, et al. $H_20_2$ producing lactobacilli and acquisition of vaginal infections. J Infect Dis 1996;174:1058-63
4. Cherpes TL, Meyn LA, Krohn MA, Lurie JG, Hillier SL. Association between acquisition of herpes simplex virus type 2 in women and bacterial vaginosis. Clin Infect Dis 2003:37;319-25.
5. Watts DH, Fazarri M, Minkoff H, Hillier SL, Sha B, Glesby M, et al. Effects of bacterial vaginosis and other genital infections on the natural history of human papillomavirus infection in HIV-1-infected and high-risk HIV-1-uninfected women. J Infect Dis. 2005:191:1129-39.

6. Cohen CR, Duerr A, Pruithinithada H, et aI. Bacterial vaginosis and HIV seroprevalence among female commercial sex workers in Chiang Mai, Thailand. AIDS1995;9:1093-7.
7. van De Wijgert JH, Mason PR, Gwanzura L, Mbizvo MT, Chirenje ZM, Iliff V, et al. Intravaginal practices, vaginal flora disturbances, and acquisition of sexually transmitted diseases in Zimbabwean women. J Infect Dis 2000;181:587-94.
8. Martin H, Richardson BA, Nyange PM, Lavreys L, Hillier SL, Chochar B, Mandaliya K, et al. Vaginal lactobacilli, microbial flora, and risk of Human Immunodeficiency Virus type I and sexually transmitted disease acquisition. J Infect Dis 1999;180:1863-8.
9. Amsel R, Totten PA, Spiegel CA, Chen KCS, Eschenbach DA, Holmes KK. Nonspecific vaginitis: diagnostic criteria and epidemiologic associations. Am J Med 1983;74:14-22.
10. Nugent RP, Krohn MA, Hillier SL. Reliability of diagnosing bacterial vaginosis is improved by a standardized method of gram stain interpretation. J Clin Microbiol. 1991;29:297-301.
